# Hypoxia Increases the Efficiencies of Cellular Reprogramming and Oncogenic Transformation in Human Blood Cell Subpopulations In Vitro and In Vivo

**DOI:** 10.3390/cells13110971

**Published:** 2024-06-04

**Authors:** Adrián Moratilla, Diana Martín, Marta Cadenas-Martín, Martha Stokking, Maria Angustias Quesada, Francisco Arnalich, Maria P. De Miguel

**Affiliations:** 1 Cell Engineering Laboratory, La Paz University Hospital Health Research Institute, IdiPAZ, 28046 Madrid, Spain; amoratillariofrio@gmail.com (A.M.); dianamartinlorenzo11@gmail.com (D.M.); cadenasm95@gmail.com (M.C.-M.); marthastokking@gmail.com (M.S.); 2 Internal Medicine Service, La Paz University Hospital, IdiPAZ, 28046 Madrid, Spain; mangustias.quesada@salud.madrid.org (M.A.Q.); farnalich@salud.madrid.org (F.A.)

**Keywords:** cellular reprograming, induced pluripotent stem cells, peripheral blood mononuclear cells, adipose-derived stem cells, hypoxia, chronic obstructive pulmonary disease

## Abstract

Patients with chronic hypoxia show a higher tumor incidence; however, no primary common cause has been recognized. Given the similarities between cellular reprogramming and oncogenic transformation, we directly compared these processes in human cells subjected to hypoxia. Mouse embryonic fibroblasts were employed as controls to compare transfection and reprogramming efficiency; human adipose-derived mesenchymal stem cells were employed as controls in human cells. Easily obtainable human peripheral blood mononuclear cells (PBMCs) were chosen to establish a standard protocol to compare cell reprogramming (into induced pluripotent stem cells (iPSCs)) and oncogenic focus formation efficiency. Cell reprogramming was achieved for all three cell types, generating actual pluripotent cells capable for differentiating into the three germ layers. The efficiencies of the cell reprogramming and oncogenic transformation were similar. Hypoxia slightly increased the reprogramming efficiency in all the cell types but with no statistical significance for PBMCs. Various PBMC types can respond to hypoxia differently; lymphocytes and monocytes were, therefore, reprogrammed separately, finding a significant difference between normoxia and hypoxia in monocytes in vitro. These differences were then searched for in vivo. The iPSCs and oncogenic foci were generated from healthy volunteers and patients with chronic obstructive pulmonary disease (COPD). Although higher iPSC generation efficiency in the patients with COPD was found for lymphocytes, this increase was not statistically significant for oncogenic foci. Remarkably, a higher statistically significant efficiency in COPD monocytes was demonstrated for both processes, suggesting that physiological hypoxia exerts an effect on cell reprogramming and oncogenic transformation in vivo in at least some cell types.

## 1. Introduction

Patients with chronic hypoxia due to various diseases show a higher tumor incidence, particularly in lung, genitourinary tract, gastrointestinal, reproductive (male and female), hematologic, breast, respiratory, and skin cancers [1,2,3,4]; however, no primary common cause has yet been recognized. Two separate mechanisms are most likely involved: (1) Several studies have suggested an immune system downregulation involving T-cells and monocytes [5,6,7,8]; (2) moreover, hypoxia appears to be directly involved in oncogenic transformation in several cancer types [9,10,11,12]. Increasing evidence points to the role of hypoxia in the emergence of cancer stem cells (CSCs) [9,13], which have been identified as immortal tumor-initiating cells that can self-renew and have been identified in a variety of solid tumors, such as colon, lung, prostate, ovary, brain, and melanoma cancers (For a concise review, see [14].).

Interestingly, cell reprogramming and oncogenic transformation are related processes. In classical oncogenic focus (OF) formation assays, fibroblast transduction with Klf4 and c-Myc promotes unlimited self-renewal in vitro and malignant fibrosarcoma formation in vivo [15]. This oncogenic transformation model is useful for easily testing the pathways involved in oncogenesis. Remarkably, this in vitro oncogenic transformation is surprisingly similar to cellular-reprogramming models. With a very similar system, cellular reprogramming has been achieved with the four ‘Yamanaka factors’ (Klf4, c-Myc, Sox2, and Oct4), promoting not only the indefinite self-renewal of fibroblasts but also the acquisition of pluripotency in vitro (both defining characteristics of induced pluripotent stem cells (iPSCs)) [16,17]. In fact, the first generations of iPSCs produced teratomas in mice [18]. Moreover, our previous data have suggested that during reprogramming, Klf4 and c-Myc induce self-renewal, whereas Oct4 and Sox2 induce pluripotency [13,19] and that the latter can be activated in cultures by hypoxic conditions that activate the glycolytic metabolism [19]. Interestingly, our data then suggested that the two main characteristics of stem cells (self-renewal and pluripotency) are regulated differentially by these two groups of factors.

Each iPSC-reprogramming factor has a role in oncogenesis: For example, Oct4 is overexpressed in seminomas, and Sox2 is amplified in lung cancer, which is an essential driver of CSCs in breast cancer and Ewing sarcoma. A large variety of human cancers express high levels of c-Myc and Klf4 (For a review, see [20].). Importantly, CSCs have been associated with a low-oxygen microenvironment [13,21,22]. Among various cancer cell types, such as breast cancer and neuroblastoma, hypoxia has been observed to induce a more immature phenotype [21,23]. One study showed that hypoxia can induce an embryonic-stem-cell-like transcriptional program, including the iPSC inducers Oct4, Sox2, c-Myc, Klf4, Nanog, and microRNA-302, in 11 cancer cell lines from the prostate, brain, kidneys, cervix, lungs, colon, liver, and breasts [24].

A direct comparison of these processes could be of significant value, given the major similarities between cellular reprogramming under hypoxic conditions and in oncogenic transformation in the following processes: the transition from oxidative phosphorylation to anaerobic glycolysis, (known as the Warburg effect) [25]; the amplification of glycolytic genes, increasing the capture and consumption of glucose and decreasing oxygen consumption [26]; metabolic plasticity [27]; the variation in mitochondrial numbers; the reduction in the number of mtDNA copies; the epithelium-to-mesenchymal transition [28]; and autophagy [29], which are all hallmarks of cancer [30,31]. The genetic profile of OF cell lines has been compared with iPSC lines, and several similarities have been found, including the overexpression of glycolytic pathway genes [15,32]. As expected, the genes related to pluripotency were more differentially expressed, as well as genes related to cellular damage. Both processes have recently been compared at the single-cell level in mouse cells [33], revealing important roles for Bcl11b and c-Myc/Atoh8. 

Given the similarities between cellular reprogramming and oncogenic transformation, and the putative role of hypoxia in promoting them, in this study, we aimed at understanding the effects of hypoxia on both processes by directly comparing them in several human cell types. This direct comparison, both in vitro and under physiological circumstances, will help in understanding the processes of cell reprogramming and oncogenic transformation and in identifying the common and divergent factors among them.

## 2. Materials and Methods

### 2.1. Episomal Vectors 

The following episomal vectors were employed: MOS (64120, Addgene, Watertown, MA, USA), which contains human Oct4 and Sox2 genes; MMK (64121, Addgene, Watertown, MA, USA), which contains human Klf4 and c-Myc; and plasmid pmax-GFP (Lonza, Basel, Switzerland), which contains the green fluorescent protein (GFP) used as a reporter gene. These vectors were grown in *Escherichia coli* DH5α cells and isolated with a commercial kit for plasmid isolation by NZYTech (NZYMiniprep; NZYTech, Lisbon, Portugal), following the protocol provided. Briefly, we prepared 15 mL culture flasks with sterile Luria–Bertani medium supplemented with selective agents, such as casein peptone (10 g/L, Schärlau Microbiology, Barcelona, Spain), yeast extract (5 g/L, Schärlau Microbiology, Barcelona, Spain), NaCl (10 g/L, Panreac, Chicago, IL, USA), and ampicillin (0.1 mg/mL). We inoculated a small aliquot of the bacterium *Escherichia coli* DH5α, harboring the recombinant plasmids, in the Luria–Bertani culture and employed the NZYMiniprep Kit (NZYTech, Lisbon, Portugal) according to the manufacturer’s instructions. We precipitated the isolated DNA by adding 10% sodium acetate and 3 times the DNA volume of pure ethanol and then centrifuged the mixture in two cycles of 30 min and 15 min each at 4 °C in a 5424 R centrifuge (Eppendorf Ibérica S. L. U., Madrid, Spain). Next, we resuspended the DNA pellet in 20 μL of distilled water. The DNA was quantified using a NanoDrop ND-1000 spectrophotometer (NanoDrop, Wilmington, DE, USA).

### 2.2. Cell Types

#### 2.2.1. PBMCs

Buffy coats from healthy anonymous donors were supplied by the Transfusion Center of the Community of Madrid. PBMCs were obtained from peripheral blood from healthy human donors or patients with COPD and were subsequently isolated by density gradient centrifugation with Ficoll-Paque Plus (GE Healthcare, Chicago, IL, USA) according to the manufacturer’s instructions. We measured the percentage of PBMCs from each sample with a BD FACSCalibur (Becton Dickinson, Franklin Lakes, NJ, USA) cytometer. The culture medium consisted of Roswell Park Memorial Institute (RPMI) 1640 medium (Lonza, Basel, Switzerland) supplemented with 10% knockout fetal bovine serum (KO-FBS) and 1% penicillin/streptomycin (Gibco, Thermo Fisher Scientific, Waltham, MA, USA).

#### 2.2.2. Mouse Embryonic Fibroblasts (MEFs)

CF1-MEFs (Cat. ASF-1202, Applied StemCell, Vancouver, BC, Canada) were employed. The culture medium consisted of Dulbecco’s Modified Eagle’s Medium (DMEM) with a high glucose concentration (4.5 g/L), GlutaMAX (862 mg/L), and pyruvate (110 mg/L) (Gibco, Thermo Fisher Scientific, Waltham, MA, USA), supplemented with 10% FBS (Gibco, Thermo Fisher Scientific, Waltham, MA, USA) and 1% non-essential amino acids (Gibco, Thermo Fisher Scientific, Waltham, MA, USA).

#### 2.2.3. Adipose-Tissue-Derived Stem Cells (ADSCs)

ADSCs were obtained from the liposuction of healthy donors and preserved in liquid nitrogen stored at the La Paz Hospital Biobank. The culture medium consisted of DMEM with a high glucose concentration (4.5 g/L), GlutaMAX (862 mg/L), and pyruvate (110 mg/L) (Gibco, Thermo Fisher Scientific, Waltham, MA, USA), supplemented with 10% FBS, 1% penicillin/streptomycin, and Fungizone (1000 U/mL) (Gibco, Thermo Fisher Scientific, Waltham, MA, USA).

### 2.3. Transfection Procedure

Various quantities of episomal vectors (from 2 to 20 µg) were assayed to achieve the maximum transfection efficiency in the three cell types, particularly in difficult-to-transfect PBMCs. Various transfection methods were employed to achieve the same purpose. For MEFs and ADSCs, both cell types were cultured a day before the transfection in 6-well plates seeded at 4 × 10^5^ cells/well, employing the TurboFect Transfection Reagent Kit (Thermo Fisher Scientific, Waltham, MA, USA) for the transfection. A total of 12 μg of the expression plasmid mixture (together with 400 μL of OPTI-MEM (Gibco, Thermo Fisher Scientific, Waltham, MA, USA) and 8 μL of the TurboFect transfection reagent (Thermo Fisher Scientific, Waltham, MA, USA) were added to each well, drop by drop. The MEF and ADSC medium were removed the day after the transfection and replaced with embryonic-stem-cell medium. Transfected MEFs and ADSCs were cultured under the same conditions as PBMCs.

For PBMCs, 12 μg of the expression plasmid mixture was electroporated (5 μg of MMK, 6 μg of MOS, and 1 μg of GFP) into 2 × 10^6^ PBMCs with the Amaxa Human Monocyte Nucleofector Kit buffer (Lonza, Basel, Switzerland) according to the manufacturer’s instructions.

### 2.4. PBMC Subpopulation Isolation

The standard Ficoll extraction method was employed to isolate PBMCs from healthy donors under sterile conditions. The separation of the leukocyte subpopulations was performed with a MACS kit specific for the separation of monocytes from other cell types, following the provided protocol (Monocyte Isolation Kit II Human, Miltenyi Biotech, Bergisch Gladbach, Germany). The efficacy of the separation was checked by flow cytometry analysis in a FACSCalibur cytometer, evaluating it with CellQuest Pro software (Becton Dickinson BioSciences, Franklin Lakes, NJ, USA).

### 2.5. Nucleofection of PBMC Subpopulations

Nucleofection was performed in an Amaxa Nucleofector, following the guidelines provided in the Amaxa Human Monocyte Nucleofector Kit (Lonza, Basel, Switzerland) and employing the programs U-008 for PBMCs and lymphocytes and Y-001 for monocytes. For iPSC generation, 5 µg of MMK and 6 µg of MOS were employed; for OFs, only 5 µg of MMK was added to the reaction mixture. In both cases, 1 µg of the GFP plasmid (Lonza, Basel, Switzerland) was also added for the transfection efficiency analysis. A total of 1.5 million cells were separated per reaction in 4 wells of 24-well plates (187,500 cells/cm^2^).

The transfection efficiency was monitored with an inverted fluorescent optical microscope (Axiovert 200, Zeiss, Oberkochen, Germany) by searching for GFP expression. Three or four days after the transfection, transfected PBMCs were plated into 24-well plates (2 × 10^6^ PBMCs) over a mitomycin-treated MEF feeder layer in embryonic stem cell (ESC) medium, containing DMEM F12 supplemented with 20% knockout-FBS, 1% penicillin/streptomycin, 2 mM non-essential amino acids, 0.1 mM β-mercaptoethanol, 20 ng/mL of FGF2, and 50 μg/mL of ascorbic acid. PBMCs were cultured at 37 °C in 5% CO_2_, either under conditions of normoxia (21% O_2_) in a Hera Cell 150 incubator (Heraeus, Hanau, Germany) or under conditions of hypoxia (3% or 5% O_2_, see below) in a Sanyo CO_2_/N_2_ MCO-18 incubator. The medium was changed daily.

### 2.6. Retroviral Transduction

A total of 9 × 10^6^ Phoenix Amphotropic 293 cells were seeded in 150-mm culture dishes at a final volume of 20 mL and incubated in a 37 °C, 5% CO_2_ incubator. The cell culture dishes were previously coated with poly-L-lysine and the plates were incubated for 1 h at room temperature (RT). The next day, Phoenix Amphotropic 293 cells were transfected with the FuGENE–DNA complex. Briefly, 0.873 mL of OPTIMEM was placed in two separate 1.5 mL tubes (one for each of the plasmids used for transfection, pMX-Oct4-Sox2-mOrange, and pMX-Klf4-c-Myc-GFP), and 54 µL of the FuGENE-6 transfection reagent was added and incubated for 5 min. A total of 20 µg of pMX-DNA plasmids were added dropwise to solutions of FuGENE–OPTIMEM and incubated for 15 min. The FuGENE–DNA solution was then added dropwise to the plates with the Phoenix Amphotropic 293 cells and returned to a 37 °C, 5% CO_2_ incubator overnight. A total of 20 mL of the viral supernatant was collected from every plate 48 h after the transfection and filtered through a 0.45 µm polyvinylidene fluoride filter to remove any residual cells, and 1 µL (10 mg/mL) of polybrene was added for each milliliter of the viral supernatant. The viral supernatant was pre-loaded onto RetroNectin-coated plates. Equal amounts of each transcription factor were pipetted into the wells to reach a total volume of 1 mL (500 µL of OCT4/Sox2 and 500 µL of Klf4/c-Myc for iPSC generation or 1 mL of Klf4/c-Myc for OF generation). The plate was set in a centrifuge pre-warmed to 32 °C and centrifuged for 1 h at 2000× *g* and 32 °C. The CD14+ cells were collected by centrifugation for 5 min at 200× *g* and RT, and the cell pellet was resuspended in RPMI complete medium, at a concentration of 8 × 10^4^ cells/mL, and incubated with the retroviruses for 12 h in a 37 °C, 5% CO_2_ incubator. Infection was repeated three times in the following manner: 500 µL from each well was removed from the 24-well plates containing CD133+ cells, and 2 × 500 µL of fresh viral supernatant (pMX-Oct4-Sox2-mOrange and pMX-Klf4-c-Myc-GFP) or 1000 µL of fresh viral supernatant (pMX-Klf4-c-Myc-GFP) was added to infect the cells a second and third time. The same day, 6-well plates were coated with Matrigel (23 µg/cm^2^) for 3 h at RT and seeded with irradiated human feeder fibroblasts (iHFF, approx. 70,000 cells/cm^2^) in DMEM supplemented with 10% FBS, 1 × penicillin/streptomycin, and 1 × Glx.

### 2.7. Sendai Virus Production and Infection

Both the CD14-positive cell fraction and CD14-negative cell fraction extracted from peripheral blood were seeded in RPMI medium supplemented with 5% FBS (RPMI complete) and cytokines (CD14+: M-CSF, GM-CSF, and IL-6; CD14-: SCF, FLT3L, IL-3, and IL-6). The medium was changed every second day. After four days of incubation, the cells were infected with a Sendai CytoTune 2.0 kit (4 factors, OSKM) overnight and seeded in RPMI complete with cytokines on Matrigel-coated dishes for two days. The medium was then changed to Repro TeSR. The medium was changed every second day, and photographs were taken every day.

### 2.8. Induced Pluripotent Stem Cell Culture

The cultured cells were followed for 2–3 weeks, which is the time estimated for the generation of iPSCs. Colonies similar to ESCs were selected for further culture and evaluation, cultured individually in 96-well plates (Corning, Corning, NY, USA) with ESC medium and ESGRO mLIF medium supplement (Leukemia Inhibitory Factor) (1000 U/mL, Millipore, Burlington, MA, USA).

Transfected cells were seeded on top of a layer of feeder mouse embryonic fibroblasts (irradiated MEFs, Millipore CF1 Embryomax, Merck Millipore, Burlington, MA, USA) at a density of 75,000 MEFs/cm^2^. The culture medium for the iPSCs was StemPro (Gibco, Thermo Fisher Scientific, Waltham, MA, USA) for the first day and TeSR2 (StemCell, Vancouver, BC, Canada) after that. The culture medium for OFs was the standard MEF medium (DMEM with 10% FBS, 1% penicillin/streptomycin, and 2 mM non-essential amino acids) for the whole experiment. The day after the transfection, the medium was only added, rather than changed, to ensure the attachment of the cells; after that, the media were changed every other day. MethoCult medium (StemCell, Vancouver, BC, Canada) was used for OF colony passages to test for attachment-free growth. The plates were kept under standard culture conditions of 37 °C, 5% CO_2_, and normoxia (21% O_2_) in a HeraCell 150 incubator (HeraCell 150, Heraeus, Hanau, Germany).

### 2.9. Alkaline Phosphatase Assay

After various time periods, the wells were assessed for the presence of iPSC or OF colonies by an alkaline phosphatase assay. Cells were fixed in 4% paraformaldehyde in phosphate-buffered saline (PBS), pH 7.4, for 20 min at RT. The alkaline phosphatase assay mixture contained 1 mg/mL of Fast Red TR (Merck, Darmstadt, Germany) and 40 μL/mL of naphthol AS-MX (Merck, Darmstadt, Germany) at pH 9.2–9.6 in distilled water. The staining allowed for the manual counting of the colonies with an inverted optical microscope (Axiovert 200, Zeiss, Oberkochen, Germany). Colonies had to have eight or more cells to be considered.

### 2.10. Immunofluorescence and Confocal Microscopy

For immunofluorescence studies, as positive controls of pluripotency, E14T mouse embryonic stem cells were cultured in embryonic stem cell medium (DMEM + GlutaMAX, 10% FBS–embryonic stem cell-qualified, 1% penicillin/streptomycin, 1% non-essential amino acids, and β-mercaptoethanol (100 mM)).

OF colonies from MethoCult cultures were included in the optimal-cutting-temperature OCT and frozen at −80 °C, subsequently obtaining 10 µm thick sections in a cryostat (CM3050 s, Leica, Wetzlar, Germany), which were mounted on silane-treated slides.

Preparations (OCT slides with OFs and coverslips with E14T cells) were permeabilized with Triton x-100 (Merck, Darmstadt, Germany) at 0.5% for 10 min at RT. The blockage solution included PBS + 1% bovine serum albumin (BSA) and 10% goat serum for 30 min at RT. The anti-leukemia inhibitory factor receptor antibody (Santa Cruz Biotechnology, Dallas, TX, USA) was diluted in PBS with 1% BSA at 1:200, and the incubation was performed overnight at RT. The secondary anti-rabbit biotinylated antibody (Vector Laboratories, Newark, CA, USA) was diluted at a concentration of 1:100 and added to the preparations after three washes with PBS + 1% BSA for 30 min at RT. For signal amplification, Avidin Texas Red was added at 1:500 for 10 min at RT in the dark.

For iPSC and OF characterizations, Oct3/4, Klf4, LIFR, and c-Myc immunofluorescences were performed. Hela or HaCat cells were used as positive controls. Anti-Oct3/4 (sc-9081, Santa Cruz Biotechnology) was employed at a 1:350 dilution, anti-Klf4 (AF3158, R&D Systems, Minneapolis, MN, USA) at a 1:100 dilution, anti-LIFR (sc-659, Santa Cruz Biotechnology) at a 1:200 dilution, and anti-c-Myc (sc-764, Santa Cruz Biotechnology) at a 1:25 dilution. Secondary antibodies, anti-rabbit FITC (Vector Laboratories) at 1:200 or anti-goat biotin and avidin–Texas Red or avidin–FITC (Vector Laboratories) at 1:500, were employed. Negative controls were performed, omitting the primary antibodies.

### 2.11. Embryonic Body Generation and Characterization

Naturally occurring embryoid bodies (EBs) were stained for markers of the three germ layers once fixed in the well at the following concentrations: anti-vimentin (mesoderm) (1:20, Dako, Santa Clara, CA, USA); anti-α-fetoprotein or anti-albumin (endoderm) (1:20, Dako, Santa Clara, CA, USA); or anti-cytokeratins (ectoderm) (1:20, Dako AE1/AE3, Santa Clara, CA, USA), with secondary fluorescent antibodies as above. Negative controls were performed similarly but omitting the primary antibodies.

The slides were mounted on Vectashield + DAPI 1:10 (Vector Labs, Newark, CA, USA) and observed under an inverted microscope (Axiovert 200, Zeiss, Oberkochen, Germany) and a confocal microscope (DMI4000 B, Leica, Wetzlar, Germany).

### 2.12. Real-Time qPCR

The total RNA was isolated from cell cultures using TRIzol reagent (Merck, Darmstadt, Germany), and 1 µg of the total RNA was employed for cDNA synthesis with the Superscript^TM^ II reverse transcriptase kit and random-hexamer primer (Invitrogen, Carlsbad, CA, USA) according to the manufacturer’s instructions. Quantitative polymerase chain reactions were set up in duplicate using PowerUp SYBR Green Master Mix (Applied Biosystems, Foster City, CA, USA) and analyzed using CFX96 Touch^TM^ real-time PCR equipment (Bio-Rad, Hercules, CA, USA). The qPCR conditions were 2 min at 50 °C for the uracil–DNA glycosylase activation, 2 min at 95 °C for the polymerase activation, followed by 40 cycles of amplification for 15 s at 95 °C or 15 s at 52, 54, or 57 °C, depending on the primers, and 1 min at 72 °C. Each gene’s expression was normalized using β2-microglobulin as the endogenous control. The respective gene expression change of each gene was calculated relative to the PBMCs as control samples using the comparative threshold cycle (Ct) method with the formula 2^−(ΔΔCt)^. Data are presented as logarithmic increments of the relative expression. The real-time PCR primer sequences were as follows: β2-microglobulin—forward: 5′-CAG GTT TAC TCA CGT CAT CCA GC-3′, reverse: 5′-TCA CAT GGT TCA CAC GGC AGG C-3′ [34]; LIFR—forward: 5′-AGG GAA GTT GGA AGG AGA TTG-3′, reverse: 5′-GGA GAA GCC ACG AAT CTA ACT G-3′ [35]; Oct3/4—forward: 5′-CCT CAC TTC ACT GCA CTG TA-3′, reverse: 5′-ACA GCC TTG TAC CCT GGT CT-3′ [36]; ESRG—forward: 5′-ACA GCC TTG TAC CCT GGT CT-3′, reverse: 5′-CAA TGG TGC GAA GCT GTG TT-3′ [37]; Klf4—forward: 5′-GCT GTG GAT GGA AAT TCG CC-3′, reverse: 5′-CTT CTG GCA GTG TGG GTC AT-3′ [38]; Nanog—forward: 5′-GTC TTC TGC TGA GAT GCC TCA CA-3′, reverse: 5′-CTT CTG CGT CAC ACC ATT GCT AT-3′ [39].

### 2.13. Data Analysis and Statistics

For the ADSCs, cells from two liposuction donors were used at passages 1–3. A total of 46 donors were used for the PBMC experiments. At least two technical replicates of each condition were performed, going up to four replicates if the cell count allowed for it, for each experiment. For the COPD versus healthy donor comparison, age-matched donors were employed to rule out differences because of age. The efficiencies for each cell type and nucleofection program were calculated with the number of GFP-positive cells per total cells for the experiments that showed a signal, to correct the reprogramming efficiency by the efficiency of the transfection.

The results are shown as means and standard deviations of all the replicates for each condition, and the differences between the means were tested with Student’s *t*-test. Comparisons between means were performed with Student’s *t*-test for non-parametric analysis, using non-paired analysis. Statistical significance was considered at *p*-values ≤ 0.05. The statistical analysis was first performed in Microsoft Excel and later corroborated with GraphPad Prism 7 (GraphPad Software, La Jolla, CA, USA). Graphics were obtained in GraphPad Prism.

## 3. Results

### 3.1. Cell Type Influences Transfection Efficiency but Oxygen Concentration Does Not

Given that hypoxia has been demonstrated to increase reprogramming efficiency in MEFs [16], they were employed as controls to compare transfection and reprogramming efficiencies. Human ADSCs were employed as a control of the same in human cells. Human peripheral blood mononuclear cells (PBMCs) were chosen because they are easy to obtain and ethically neutral. A standard protocol was thereby established in which a comparison of the reprogramming efficiency in normoxia versus hypoxia and between the various types of patients was possible. In particular, the initial aim was to establish whether there were differences in the transfection efficiency between cell types.

To this end, various episomal quantities (from 2 to 20 µg) and buffers were employed for nucleofection, with nucleofector programs aimed at the maximum transfection efficiency for each cell type. The optimal episomal DNA transfection quantity was 12 µg of plasmid DNA. There were significant differences with respect to the cell type, with MEFs having the highest efficiency and PBMCs having the lowest, as expected (Supplemental Figure S1A).

The influences of normoxic and hypoxic conditions on the transfection efficiency were also investigated. The reprogramming of the three cell types was performed under three metabolic conditions: normoxia (21% O_2_), hypoxia (3% O_2_) on the same day of the transfection (at day 0 post-transfection), and hypoxia (3% O_2_) at day 4 post-transfection. These last two conditions were chosen to elucidate whether hypoxic conditions influence only reprogramming once it has already started or are favorable only if hypoxia occurs prior to reprogramming. No statistically significant differences were found in the transfection efficiency with respect to oxygen concentration conditions (Supplemental Figure S1B), thus allowing for the comparison of the reprogramming efficiency for each cell type under various oxygen concentration conditions (see below).

### 3.2. Cell Reprogramming Was Efficiently Achieved for All Three Cell Types

**Figure 1:**
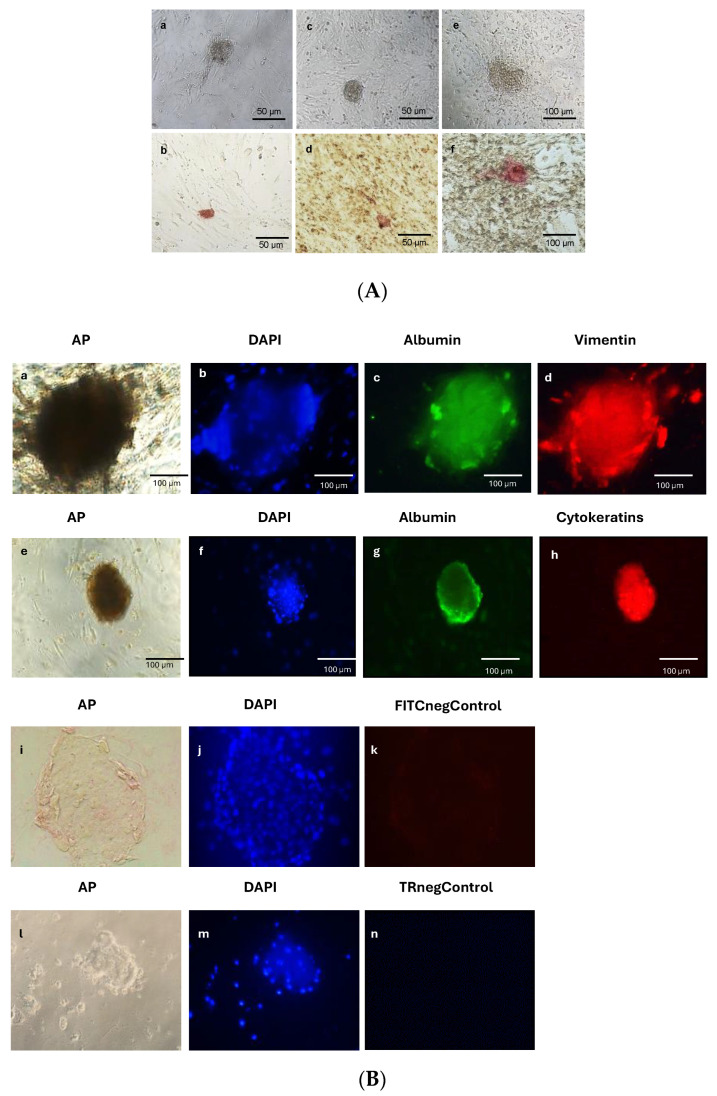
Reprogrammed cell colonies are pluripotent. (**A**) Phase contrast microscopy (**a**,**c**,**e**) and alkaline phosphatase (AP) staining (**b**,**d**,**f**) of reprogrammed cell colonies from PBMCs (**a**,**b**), ADSCs (**c**,**d**), and MEFs (**e**,**f**). (**B**) In vitro embryoid body (EB) formation and characterization of reprogrammed cells by immunofluorescence for markers of the three embryonic layers. (**a**–**d**,**i**–**k**): Reprogrammed cells from MEFs. (**e**–**h**,**l**–**n**): Reprogrammed cells from PBMCs. (**i**–**n**): Negative controls for secondary antibodies showing no staining (FITC negative control and Texas Red negative control). EBs are positive for several embryonic layers (albumin for endoderm, vimentin for mesoderm, and cytokeratins for ectoderm). 4’,6-Diamidino-2-phenylindole (DAPI) was employed to stain cell nuclei. Colonies are now negative for AP, given that differentiation has occurred. Bars: 50 or 100 μm, as indicated.

Despite the low transfection efficiency, cellular reprogramming by episomal vectors was possible for all three cell types employed and was achieved at very high efficiencies. The mean efficiency was 0.18% ± 0.04%, and the maximum efficiency was 0.48% ± 0.1%. Examples of reprogrammed cell colonies from the various cell types used are shown in Figure 1A.

### 3.3. The Generated Reprogrammed Cells Are Pluripotent

The generated colonies were pluripotent, given that they had the potential to differentiate into cell types of the three embryonic layers in vitro after the spontaneous formation of embryoid bodies (Figure 1B).

### 3.4. Metabolic Conditions Have an Effect on the Reprogramming Efficiency

**Figure 2:**
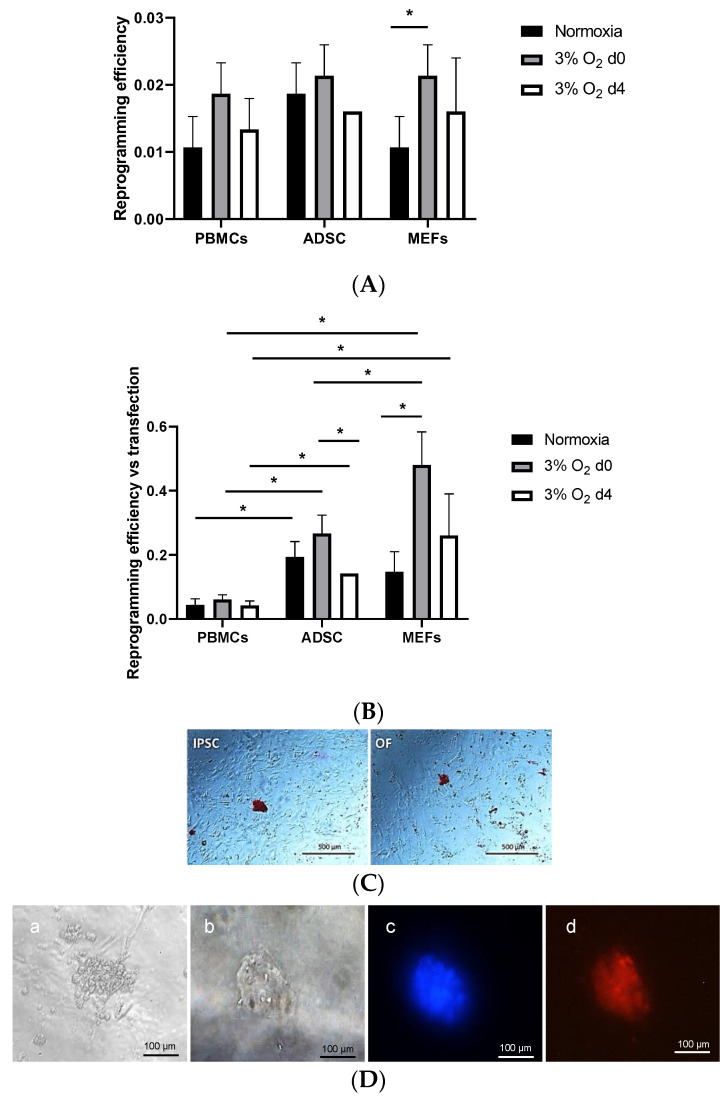
(**A**) Effects of various oxygen concentrations and timings on cell reprogramming. Normoxia (black bars), 3% O_2_ from day 0 of reprogramming (gray bars), and 3% O_2_ from day 4 (white bars). The asterisk denotes significant differences at *p* ≤ 0.05. (**B**) Reprogramming efficiency with respect to transfection efficiency at various oxygen concentrations in cell reprogramming of the three cell types used. Normoxia (black bars), 3% O_2_ from day 0 of reprogramming (gray bars), and 3% O_2_ from day 4 (white bars). Asterisks denote statistically significant differences at p ≤ 0.05. (**C**) Pluripotency is associated with alkaline phosphatase expression, which is revealed with Fast Red. The iPSCs and OFs were successfully generated from blood cells from the same donor. (**D**) (**a**) Phase contrast microscopy of oncogenic focus growing without anchorage in MethoCult. (**b**–**d**) Immunohistochemistry of oncogenic focus (growing attached) for leukemia inhibitory factor receptor (**d**, in red) (c: nuclei in blue with DAPI) and (**b**) OF in phase contrast. Bars: 100 μm.

The effect of hypoxic conditions on the reprogramming efficiency was analyzed for the three cell types. Hypoxia slightly increased the reprogramming efficiency in all three cell types and was significant for MEFs (Figure 2A).

To ensure even comparisons in reprogramming efficiency between cells showing differences in transfection efficiency, the transfection efficiency for each cell type was considered; from here on, all the efficiencies are shown with respect to the transfection efficiency. The hypoxia effect was maintained for MEFs, and higher differences arose between cell types (Figure 2B).

### 3.5. Cell Reprogramming and Oncogenic Focus Formation Can Be Achieved from the Same PBMCs

First, OF lines were established from PBMCs from healthy donors. For this purpose, OFs were derived by nucleofection with c-Myc and Klf4 factors, while iPSCs were derived from the same cells from the same donor with the addition of Oct4 and Sox2 to the other two factors. The colonies obtained were, in all the cases, alkaline-phosphatase-positive (Figure 2C). No morphological differences were found between the two types of colonies.

**Figure 3:**
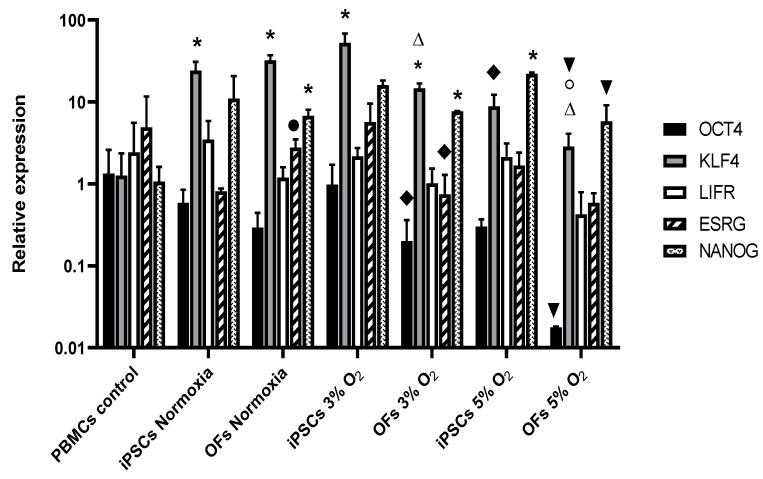
Quantitative RT-PCR for pluripotency genes after iPSC and OF derivations from PBMCs under different metabolic conditions. Data are expressed as expressions relative to β2-microglbulin. Significant differences at *p* ≤ 0.05 are as follows: *: compared with PBMCs; ●: compared with iPSCs derived in normoxia; Δ: compared with OFs derived in normoxia; ♦: compared with iPSCs derived in 3% O_2_; ○: compared with OFs derived in 3% O_2_; ▼: compared with iPSCs derived in 5% O_2_.

**Figure 4:**
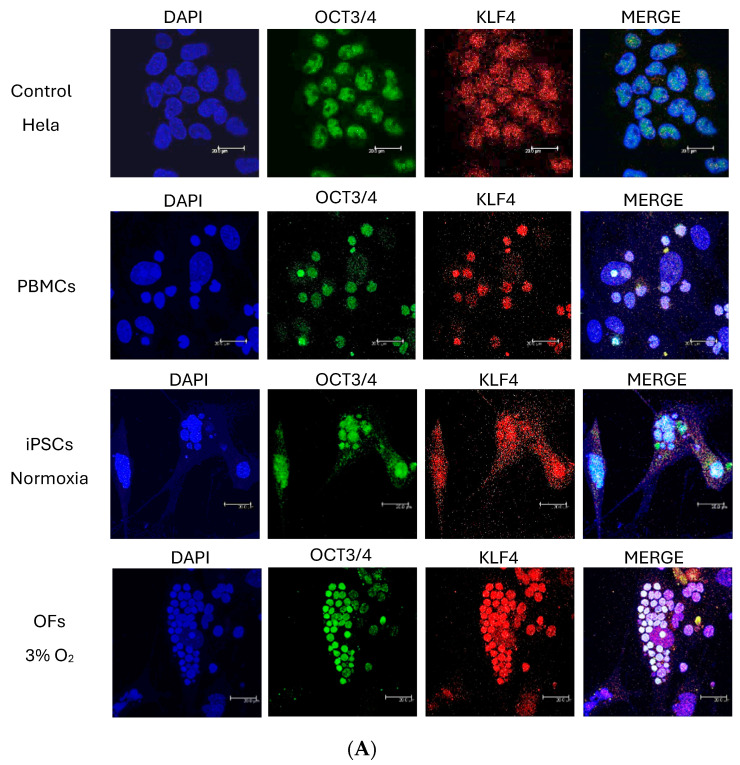

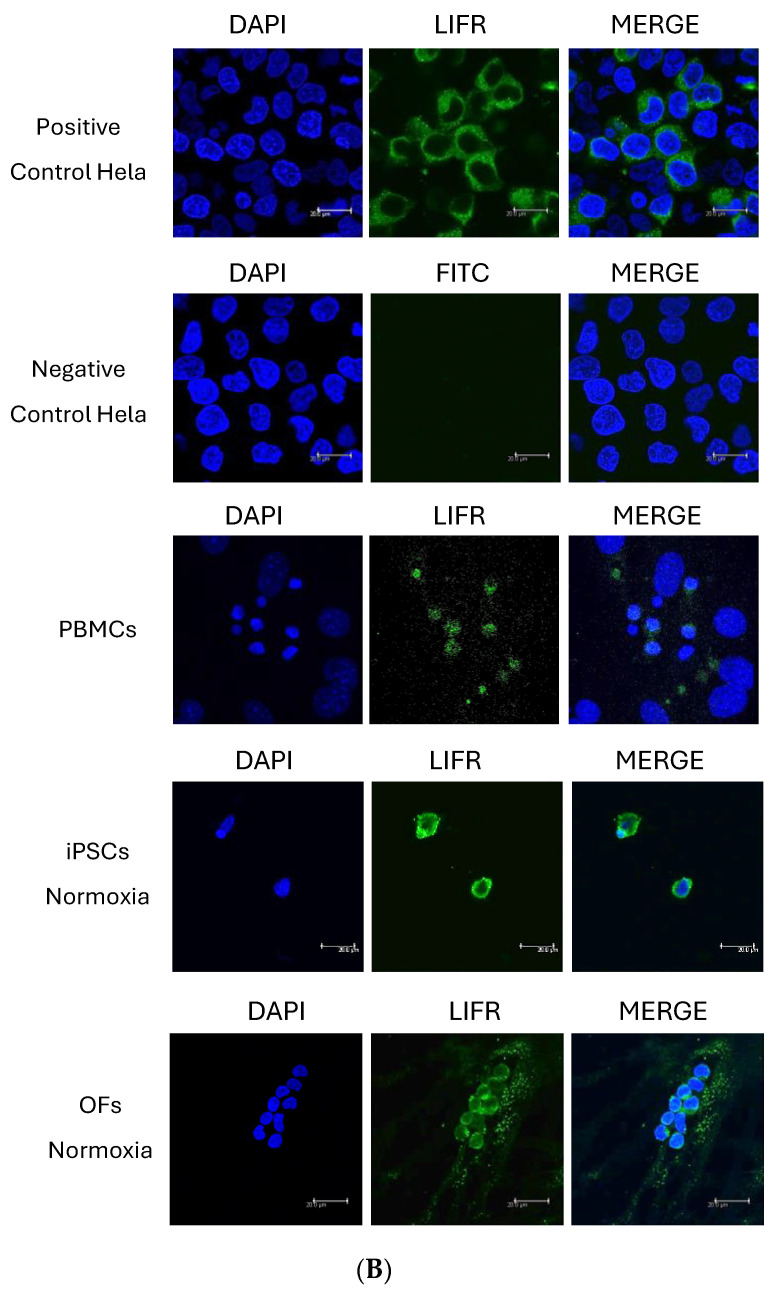

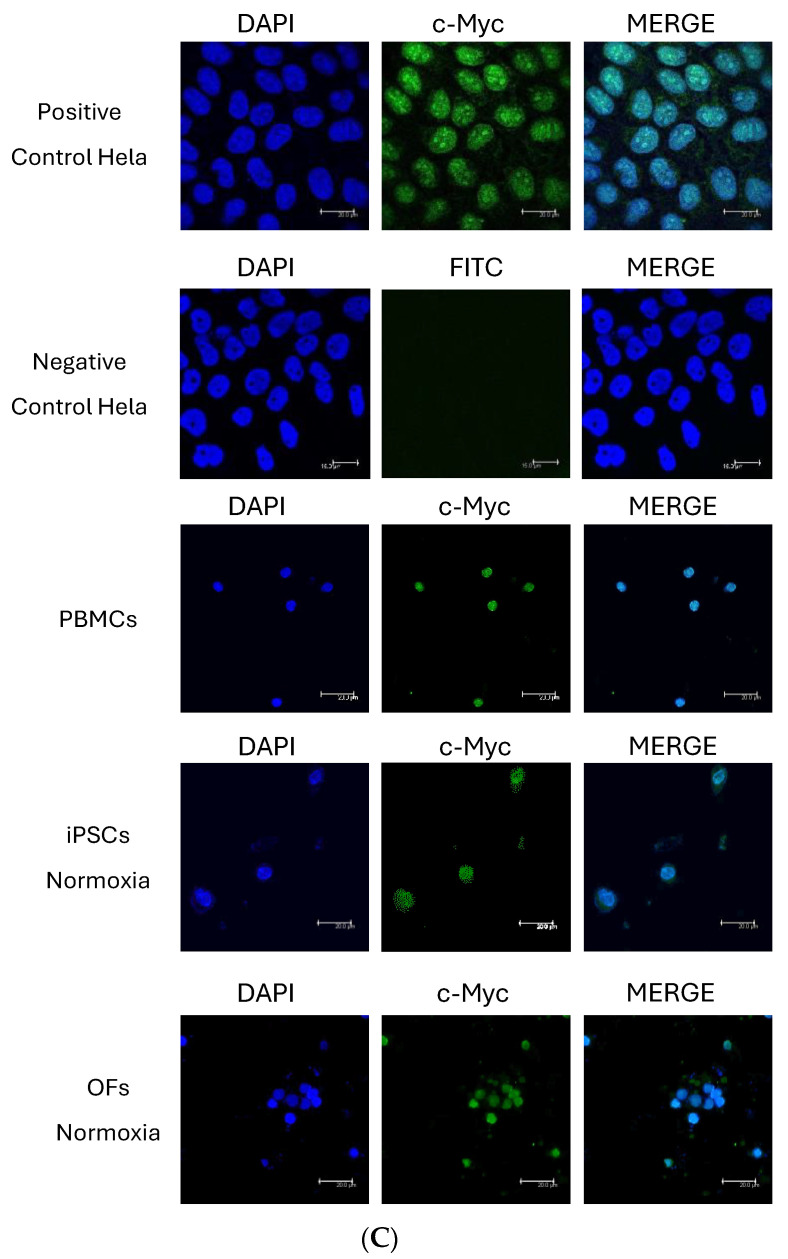
Characterization of iPSC and OF protein markers by immunofluorescence. (**A**) Oct3/4 (FITC, green) and Klf4 (TR, red) colocalization in the cells’ nuclei. (**B**) LIFRβ (FITC, green) in the cells’ cytoplasms. Hela cells in the first row as positive controls and in the second one as negative controls. (**C**) c-Myc (FITC, green) labelling in the cells’ nuclei. Hela cells in the first row as positive controls and in the second one as negative controls. Cell nuclei were stained with 4’,6-Diamidino-2-phenylindole (DAPI). Bars: 200 μm.

To verify that the generated OF lines were actual OFs, the OF lines were cultured in non-contact culture medium (MethoCult), a unique characteristic of transformed cells (Figure 2D). The OFs were also found to be positive for the leukemia inhibitory factor receptor, effectively proving their identity as oncogenic transformed cells (Figure 2D). In addition, the characterization of iPSCs and OFs was performed by means of qPCR for markers Oct4, Klf4, LIFRβ, ESRG, and Nanog (Figure 3) and immunocytochemistry and confocal microscopy for markers Oct4, Klf4, LIFRβ, and c-Myc (Figure 4). With PBMCs, the main differences were increased Klf4 expression at 3% O_2_ but not at 5% (Figure 3). Surprisingly, when comparing iPSC derivation in normoxia versus 3% and 5% O_2_, however, there were no differences because of the high standard deviation. Nevertheless, Klf4 levels were significantly lower at 5% O_2_ than at 3% O_2_. LIFRβ and ESRG levels did not differ by oxygen concentration because of the high standard deviation. Nanog levels were significantly higher in the iPSC derivation at 5% O_2_ when compared with the control PBMCs but showed no differences at 3% O_2_ or in normoxia, suggesting that iPSC derivation implies an increase in Nanog levels. When comparing OF derivations under differing metabolic conditions, Nanog expression levels were significantly higher in OFs in normoxia and at 3% O_2_ (but not at 5% O_2_) compared with PBMCs, although there were no statistically significant differences between 3% and 5%, suggesting that a high standard deviation could have accounted for this result. The tendency was, once again, increased Nanog levels under all the conditions inducing OF derivation. When comparing iPSC versus OF derivations under the same metabolic conditions, ESRG levels were significantly higher in the normoxia-derived OFs. At 3% O_2_, Oct4 and ESRG levels were significantly lower in OFs; at 5% O_2_, lower Oct4, Klf4, and Nanog levels were found (Figure 3).

### 3.6. Comparison between Reprogramming and Oncogenic Transformation Efficiencies

**Figure 5:**
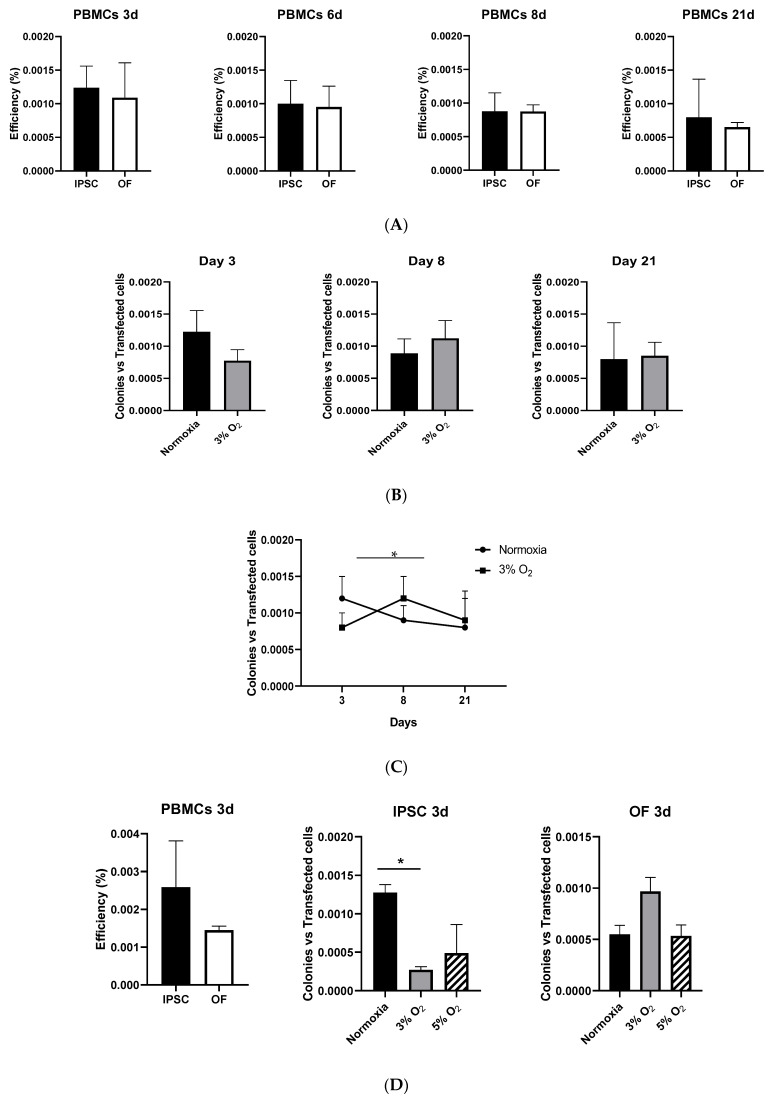
(**A**) Comparison of reprogramming efficiency (to iPSCs) versus transformation efficiency (OFs) of human peripheral mononuclear blood cells (PBMCs) from healthy donors 3, 6, 8, and 21 days after nucleofection under normoxic conditions. No statistically significant differences were observed at *p* ≤ 0.05. Data are shown in percentages with respect to the transfection efficiency. (**B**) Comparative efficiencies of iPSC formation in normoxia (black bars) versus 3% O_2_ (gray bars) on different days from healthy donor PBMCs. (**C**) Kinetics of reprogramming. There was a statistically significant difference at 3% O_2_ between days 3 and 8 (asterisk at *p* ≤ 0.05). (**D**) Comparison of reprogramming efficiencies at 20% versus 5% versus 3% oxygen levels. No differences between the reprogramming efficiency (iPSCs) versus the transformation efficiency (OFs) of human PBMCs from healthy donors 3 days after nucleofection were encountered at 5% O_2_ levels (**left**). Comparative efficiencies of iPSC formation in normoxia versus 3% O_2_ or 5% O_2_ at day 3 from healthy donor PBMCs (**center**). There was a statistically significant difference in the reprogramming efficiency only at 3% O_2_ at day 3 compared with normoxia (asterisk at *p* ≤ 0.05). No differences in the OF derivation efficiency (**right**) were observed. Data are from *n* = 2 donors × 2 technical replicates.

The efficiencies of reprogramming and oncogenic transformation in human PBMCs were then compared, finding no statistically significant differences between the efficiencies of these two processes (Figure 5A). A tendency for certain colonies to disappear over time was observed. The absence of significant differences in the efficiency of these processes could help when comparing patients and healthy donors.

### 3.7. Comparison of Cell Reprogramming Kinetics with Respect to Metabolic Conditions

Regarding the comparison of the reprogramming efficiency in normoxia and at 3% O_2_ in PBMCs (Figure 5B), the reprogramming efficiency of iPSCs obtained from PBMCs was between 0.07% and 0.1%. There were no statistically significant differences between normoxia and 3% O_2_ in the reprogramming efficiency of iPSCs obtained from PBMCs on different days. However, the reprogramming kinetics varied: whereas the reprogramming efficiency in normoxia decreased between days 3 and 8, the efficiency increased in 3% O_2_, with a statistically significant difference in the reprogramming efficiency (Figure 5C).

While reviewing this article, it was pointed out that physiological blood oxygen levels are lower than 20% and could be affecting the results [40]. Comparative experiments were, therefore, performed at 20%, 5%, and 3% oxygen levels. The results show that 5% oxygen did not result in differences between the reprogramming efficiency (to iPSCs) versus the transformation efficiency (OFs) of human PBMCs from healthy donors 3 days after nucleofection (Figure 5D). In comparison with 3% O_2_, no differences were encountered at day 3 in either the reprogramming efficiency (iPSC) or oncogenic transformation (OF) (Figure 5D). As stated above, however, there were differences at the mRNA level (Figure 3) and in PBMC subpopulations (see below).

### 3.8. Reprogramming and Oncogenic Focus Formation from Cell Subtypes within Peripheral Blood

The hypothesis was that the absence of statistical significance in the cell reprogramming efficiency under hypoxic conditions with respect to normoxia in PBMCs could be because of the presence of various cell types that might respond to hypoxia differently. Given that the percentage of lymphocytes is approximately 80% in peripheral blood, these findings probably reflect the lack of the influence of hypoxia on the lymphocyte reprogramming efficiency. In fact, it has been reported that monocytes are the most susceptible to metabolic changes induced by environmental conditions. The two main subtypes of PBMCs, lymphocytes and monocytes, were, therefore, isolated (Supplemental Figure S2) and reprogrammed separately.

#### 3.8.1. Generation of Reprogrammed Cells from Peripheral Blood Lymphocytes and Monocytes

**Figure 6:**
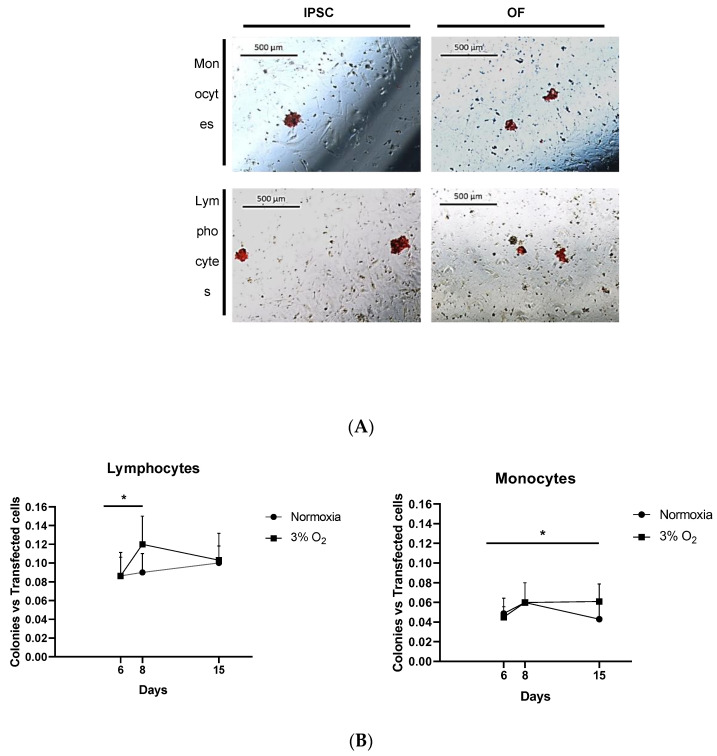
(**A**) Histochemistry for alkaline phosphatase (in red) of reprogrammed and transformed colonies; iPSCs and OFs were successfully generated from monocytes and lymphocytes from the same donor. (**B**) Kinetics of lymphocyte and monocyte subpopulation reprogramming under normoxic and 3% O_2_ conditions. Asterisks: statistically significant differences at *p* ≤ 0.05.

Both subpopulations were successfully reprogrammed and transformed (Figure 6A). The colonies had similar aspects, regardless of their origin.

As for PBMCs, the transfection efficiency in blood cell subpopulations was calculated as the number of green fluorescent protein-positive cells relative to the number of seeded cells. The transfection efficiency for the different subpopulations was as follows: lymphocytes 7% ± 4%, 22 donors; monocytes 12% ± 8%, 27 donors.

#### 3.8.2. Reprogramming Kinetics in Various Blood Subpopulations

As expected, the reprogramming kinetics showed differences in PBMC subpopulations at 3% O_2_: in the lymphocytes, there was a statistically significant difference in reprogramming efficiencies at 3% O_2_ between days 6 and 8, whereas in the monocytes, there was a statistically significant difference in reprogramming efficiencies at 3% O_2_ between days 6 and 15 (Figure 6B). No differences were encountered in normoxia.

#### 3.8.3. Cell Reprogramming and Oncogenic Transformation Efficiencies in Various Human Peripheral Blood Subpopulations

**Figure 7:**
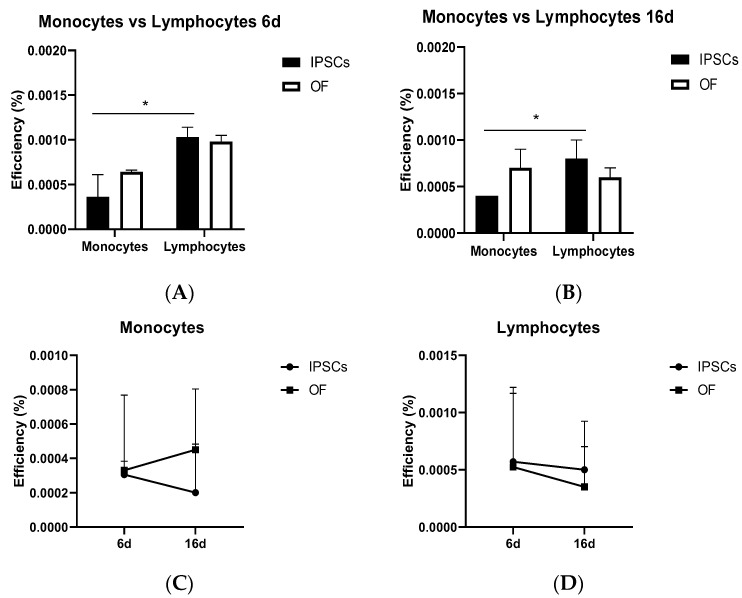
Comparative reprogramming efficiency (black bars) and oncogenic transformation efficiency (white bars) in blood cell subpopulations (monocytes and lymphocytes) from healthy donors at day 6 (**A**) or day 16 (**B**). Statistically significant differences were found between monocyte and lymphocyte reprogrammings (asterisks at *p* ≤ 0.05). No statistically significant differences were found between efficiencies of reprogrammings (circles) and oncogenic transformations (squares) (**C**,**D**); *n* = 3 donors and 4 technical replicates.

The reprogramming efficiency of lymphocytes was higher (0.08–0.1%) than that of monocytes (0.05%) on different days, with statistically significant differences (both at day 6 (Figure 7A) and at day 16 (Figure 7B)). Regarding the oncogenic transformation, the lymphocytes showed no statistically significant differences (Figure 7A,B). There were also no differences in the efficiency over time (Figure 7C,D).

**Figure 8:**
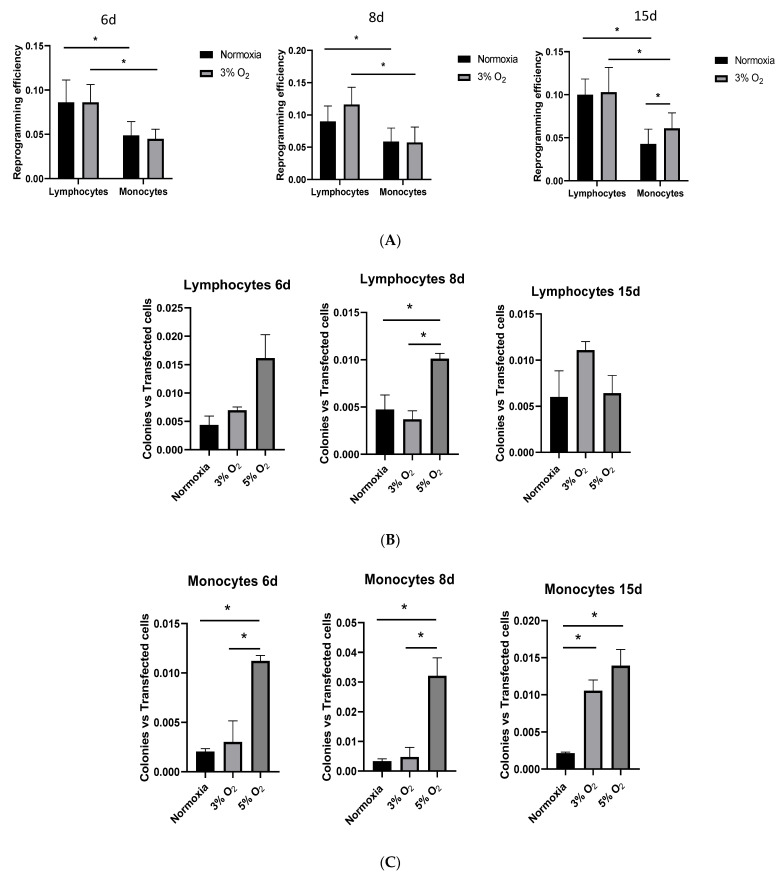
(**A**): Comparison of reprogramming efficiencies on different days after nucleofection (days 6, 8, and 15) and under normoxia and 3% O_2_ conditions. (**B**,**C**): Comparison of reprogramming efficiencies on different days after nucleofection (days 6, 8, and 15) and under normoxia or 3% O_2_ or 5% O_2_ conditions both for lymphocyte (**B**) and monocyte (**C**) subpopulations. Asterisks: Statistically significant differences at *p* ≤ 0.05.

An important finding demonstrating that the data obtained with the PBMC mixture masked the results was the statistically significant difference between normoxia and 3% O_2_ in the reprogramming efficiency of monocytes at day 15 (Figure 8A). Furthermore, the 5% O_2_ kinetics were even faster than those at 3% O_2_, with lymphocyte reprogramming being statistically significant at day 8 and, remarkably, the monocyte reprogramming efficiency being significantly higher at days 6, 8, and 15 (Figure 8B,C).

**Figure 9:**
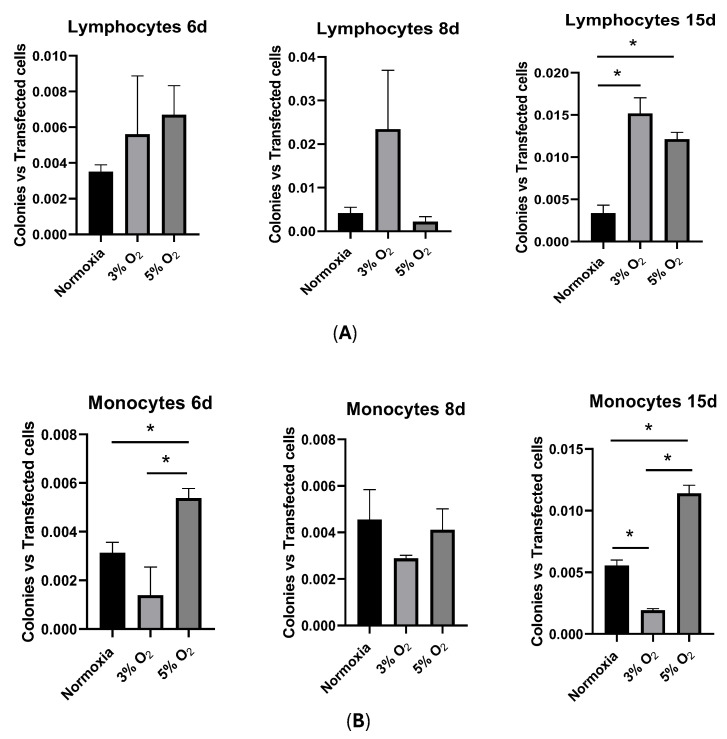
Comparison of oncogenic transformations on different days after nucleofection (days 6, 8, and 15) and under normoxia or 3% or 5% O_2_ conditions both for lymphocyte (**A**) and monocyte (**B**) subpopulations. Asterisks: Statistically significant differences at *p* ≤ 0.05.

With respect to OFs, the lymphocytes showed higher efficiencies toward oncogenic transformation at day 15 both at 3% and 5% oxygen, and monocytes showed a higher efficiency at day 6 at 5% oxygen (Figure 9).

### 3.9. Derivation of Reprogrammed Cells and Oncogenic Foci from Patients with Chronic Obstructive Pulmonary Disease

**Figure 10:**
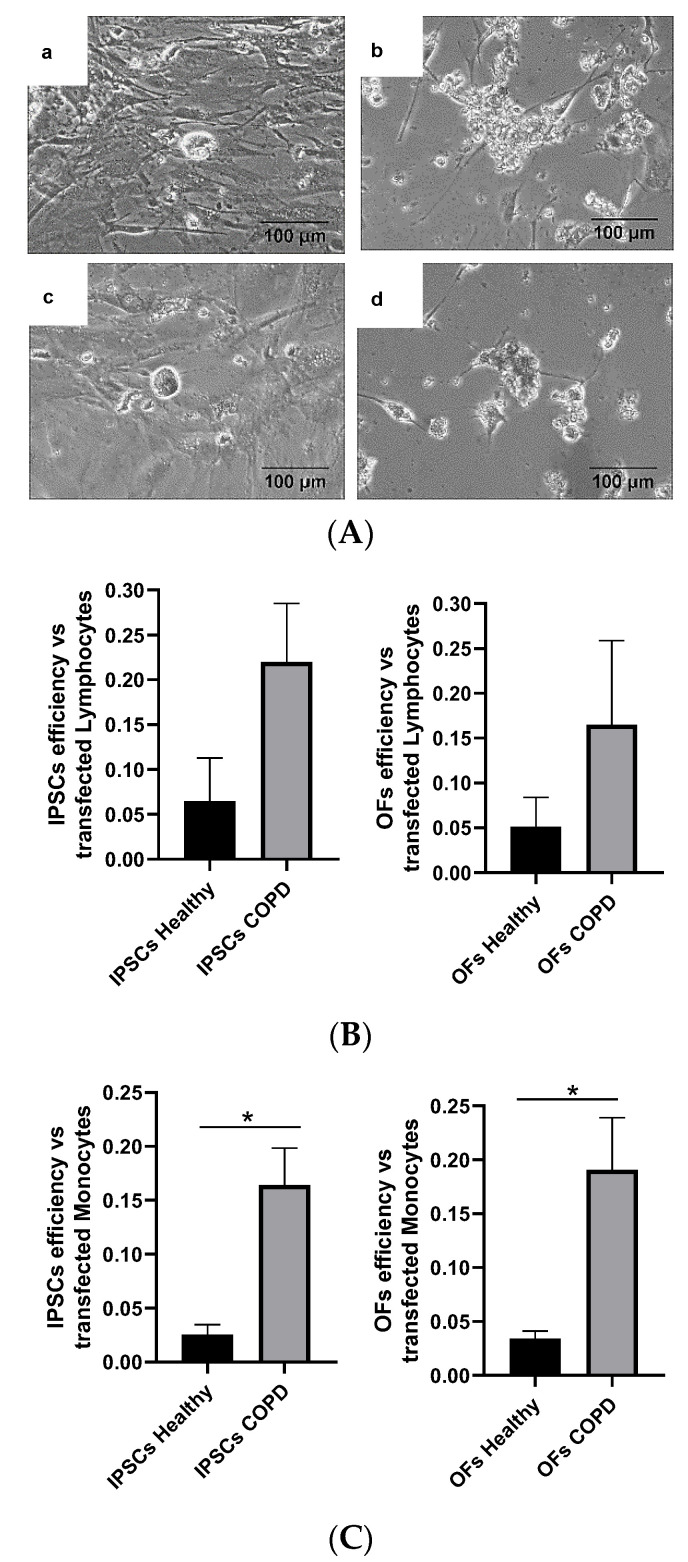
Reprogrammed cells from patients with COPD. (**A**) (**a**) Phase contrast microscopy of OFs from lymphocytes; (**b**) iPSCs from lymphocytes; (**c**) OFs from monocytes; (**d**) iPSCs from monocytes. (**B**) Comparison of cell reprogramming and oncogenic transformation efficiencies in lymphocytes from patients with COPD compared with healthy donors. (**C**) Comparison of the efficiencies of cell reprogramming and oncogenic transformation in monocytes from patients with COPD compared with healthy donors. Asterisks: Statistically significant differences at *p* ≤ 0.05.

The previous findings prompted an examination as to whether such differences in reprogramming and OF formation, because of hypoxic conditions, had a translation in vivo. To test this hypothesis, iPSCs and OFs were generated from lymphocytes and monocytes from the peripheral blood of patients with COPD (Figure 10A).

### 3.10. Efficiencies of Reprogramming and Oncogenic Transformation in Patients with COPD Relative to Healthy Donors

The iPSCs and OFs were obtained exclusively under normoxic conditions, and comparisons were performed 14 days after culturing.

With respect to the lymphocyte subpopulation, there were statistically significant differences in cell reprogramming, which were higher in the patients with COPD (Figure 10B). Similarly, there was a higher efficiency in COPD lymphocytes in the case of oncogenic transformation, although it was not statistically significant (Figure 10B).

The efficiencies of the cell reprogramming and oncogenic transformation were then compared using the blood subpopulation of monocytes. Remarkably, higher efficiency in COPD was demonstrated for both processes, with statistically significant differences (Figure 10C), suggesting that physiological hypoxia, in fact, exerts effects on cell reprogramming and oncogenic transformation in vivo, at least in certain cell populations, such as lymphocytes and monocytes.

These findings prompted us to establish reprogrammed cell lines from monocytes from healthy donors and patients with COPD, which would allow for further molecular comparison of these processes under in vivo hypoxic conditions.

### 3.11. No Establishment of Reprogrammed Cell Lines from the Monocyte Subpopulation

Employing our method of the nucleofection of episomal vectors, it was not possible, however, to establish iPSC lines either from peripheral blood monocytes of healthy donors or from patients with COPD. The following alternative methods were, therefore, employed:

Lentiviral vectors: Unfortunately, the infection of CD14+ monocytes with retroviral vectors similarly resulted in the generation of reprogrammed colonies (Supplemental Figure S3A) but which did not establish as cell lines. This occurred for monocytes from patients with COPD and those from healthy donors.

We then used the Sendai virus, which has been demonstrated to be effective for iPSC generation from PBMCs; however, no stable colonies were obtained from young-healthy-donor monocytes (Supplemental Figure S3B). CD14-positive cells showed aggregates of cells, without an iPSC-like morphology, which grew slightly and then died after a few days (Supplemental Figure S3B). CD14-negative cells (most likely lymphocytes) were employed as controls for reprogrammed cell-line generation. The first colonies arose from CD14-negative cells 8 days after infection (Supplemental Figure S3C), and 12 iPSC clones were raised.

In conclusion, although monocyte iPSC reprogramming was favored by hypoxia both in vitro and in vivo, no complete reprogramming was achieved by the use of Yamanaka factors alone.

## 4. Discussion

At the molecular level, OF formation can be considered as a partial reprogramming, given that it requires only two of the four Yamanaka factors (c-Myc and Klf4 but not Oct4 or Sox2). Evidence has shown that numerous pathways that are classically associated with oncogenesis might also regulate normal stem cell self-renewal (For reviews, see [41,42].). A direct comparison in mouse cells has recently identified two genes central to these processes: Bcl11b and c-Myc/Atoh8 [33].

When considering the cell source for reprogramming, easily accessible somatic cells are preferable. This study, therefore, employed blood cells, which supplied an effective source of iPSCs and OFs. Furthermore, PBMCs can be used immediately after extraction and do not require an expansion time and passages in cultures. However, reprogramming efficiencies and kinetics vary among somatic cell types; for example, a 100-fold higher reprogramming efficiency (0.8%) in keratinocytes than in skin fibroblasts was observed under the same conditions [43]. Moreover, the choice of the reprogramming factors also appears to be dependent on the somatic cell type. In addition to the four OSKM transcription factors described by Takahashi and Yamanaka (2006) [16] and the alternative combination (described by the Thomson group) containing Sox2, Oct4, Lin28, and Nanog [44], other transcription factors, small molecules, microRNA, and culturing conditions have been found to increase the reprogramming efficiency and iPSC quality [43].

The findings from our study showed that iPSCs can be generated from human ADSCs, PBMCs, and MEFs by the episomal vector transfection of the same four transcription factors. The reprogramming efficiency with respect to transfected cells showed a statistically significant difference between mouse and human cells, with mouse cell efficiency being higher than that of human cells, as has been previously shown [17,18]. Differences between the two groups of human cells were not statistically significant, except under hypoxia conditions, in which ADSCs showed higher reprogramming efficiency than PBMCs. These findings suggest that the fundamental transcriptional network governing pluripotency induction is common in humans and mice, although extrinsic factors and signals maintaining pluripotency are unique for each species [17].

Reprogramming efficiency is linked to the techniques employed for the reprogramming process and the cell of origin, covering a wide range, from the 0.02% initially described for MEFs [16] and human fibroblasts [17] by retroviral transduction to an impressive 24% obtained for mouse myeloid progenitors [45]. Attempts have been made to derive iPSCs more safely, such as through the use of adenoviral vectors [46], expression plasmids [47], episomal vectors [44], or even recombinant proteins [28]. These methods eliminate the problems related to the integration of exogenous genes into the genome; in return, however, they are much less efficient, reaching a maximal efficiency of 0.001% with adenoviral vectors and 0.003% with episomal vectors [48]. The efficiency of the iPSC generation by plasmids is even lower, at less than 0.0002%, which is at least 1000-fold lower than that of viral induction [49] and similar to what occurred in our study, in which reprogramming efficiencies, in most cases, were approximately 0.0001% with respect to total cells, obtaining a reprogramming efficiency of 0.182 ± 0.044% with respect to transfected cells. From a practical point of view, this efficiency is sufficiently high, given that multiple iPSC clones can be obtained from a single experiment/patient.

Overall, our efficiency results are in keeping with those expected for the episomal vector method and PBMCs [50]; however, the kinetics for the reprogramming were much faster than with other protocols, in which colonies take from 10 to 30 days to appear in cultures [51,52]. In our experiments, however, colonies with more than eight cells became alkaline-phosphatase-positive by day 3 after nucleofection, which could be because of the episomal vector methods being non-integrative. It is, therefore, unnecessary to wait for the cell to divide, whereas in integrative methods, at least one cell division is necessary for them to integrate into the genome, thereby reprogramming factor expressions [43]. This finding could also be because of the use of nucleofection, given that making pores in the cell membrane would favor the rapid and effective entry of episomal vectors. It could also simply be the case that these authors’ [51,52] threshold for considering a colony is higher than our threshold.

Although there were no statistically significant differences in the efficiency for reprogramming with PBMCs at various time points, the colonies showed a tendency to increase in size and reduce in number with time, which is probably due to the fact that certain small colonies cannot proliferate fast enough and perish or detach during the frequent medium changes, probably because of incomplete reprogramming. Three main phases have been shown to occur during iPSC derivation, each driven by characteristic genes: initiation, maturation, and stabilization [53]. Many of these earlier colonies could have undergone the initiation and maturation phases of cell reprogramming but might not have reached the stabilization phase, as previous studies from our laboratory have shown [19]. In addition, a number of colonies began to show signs of spontaneous differentiation (such as turning darker and much more compact) during the most prolonged culture periods, which could also account for the loss of AP+ colonies. The presence of markers from the three germ layers in our colony-derived EBs demonstrates that the reprogramming process had been successful and that the cells could potentially form teratomas in vivo, which is a hallmark of pluripotency. On the other hand, the ability for attachment-free growth and the presence of the leukemia inhibitory factor receptor in our OF colonies can be interpreted as a signal of the malignization of the cells, given that many tumor types show poorer prognoses when this marker is present [54]. However, other functional assays should be performed to ensure that the process that those colonies have undergone is an oncogenic transformation.

Both the lymphocyte and monocyte subpopulations achieved final reprogrammings with very similar efficiencies, which contradicts the findings of previous studies on the matter, such as those by Loh et al. and Seki et al. [51,55], in which the reprogramming efficiency of the T-cell population was 100-fold higher than that of PBMCs and where most of the colonies obtained from PBMCs proceeded from the lymphoid line. However, this difference could be because of the employed methods because both studies used a viral-based method, whereas ours employed nucleofection and episomal vectors. The fine tuning of the nucleofection program and system in our study could have accounted for our success. Monocytes appear to be more refractive to viral transduction than lymphocytes; however, with the nucleofection method adjusted to each cell type, those difficulties are overcome, and the efficiencies of both populations reach more comparable levels. There was a significant increase in the reprogramming efficiency of lymphocytes between days 6 and 8 and, in the case of monocytes, between days 6 and 15 under 3% O_2_ conditions, suggesting that 3% O_2_ progressively increases the reprogramming efficiency of iPSCs in these cells. The difference in the kinetics between monocytes and lymphocytes could be due to the fact that cell reprogramming follows an initially stochastic model that implies that the time between the various phases differs between cells [16,53]. A longer incubation time might be necessary to allow for more monocytes to be reprogrammed into a pluripotent state.

Hypoxia appears to have a favorable effect on reprogramming. In studies with human dental pulp cells exposed to 3% oxygen during the first 6 days of reprogramming, the formation of iPSC colonies increased [56]. A study by Yoshida et al. (2009) [57] observed that hypoxia increases the reprogramming efficiency in fibroblasts. Despite these findings, our results showed no statistically significant difference in reprogramming efficiencies in vitro when normoxia and hypoxia were compared, either in PBMCs or in lymphocytes. These results are logical, considering that 70–90% of PBMCs are lymphocytes [58]. However, we observed a statistically significant difference in monocyte reprogramming efficiencies after 15 days at 3% O_2_ and already after 6 days at 5% O_2_, which could be because of monocytes’ energy metabolism. Monocytes obtain energy by both oxidative phosphorylation and glycolysis, whereas lymphocytes obtain energy only through oxidative phosphorylation [59,60]. These metabolic differences between cell populations could be the cause of the higher efficiency for reprogramming in hypoxia with respect to normoxia only in monocytes.

Atkuri et al. [40] showed that culturing primary T-cells at 20% oxygen significantly alters the intracellular redox state, whereas culturing at 5% oxygen maintains the intracellular redox environment close to its in vivo status. In our experiments, 3% O_2_ versus 5% O_2_ showed that the 5% O_2_ kinetics were even faster than those at 3% O_2_, with increased lymphocyte reprogramming efficiency being significant at day 8 and, remarkably, the increased monocyte reprogramming efficiency being significantly higher already at day 6. With respect to OFs, lymphocytes showed higher efficiencies toward oncogenic transformation at day 15 both at 3% and 5% oxygen, and monocytes, already at day 6 at 5% oxygen.

We also compared 20% vs. 5% and 3% oxygen levels in iPSCs and OFs at mRNA levels by means of quantitative PCR. The main differences were in increased Klf4 expression at 3% O_2_ but not at 5% with respect to PBMCs, both for iPSCs and OFs. Surprisingly, when comparing iPSC derivations in normoxia versus at 3% and 5% O_2_, there were no differences in any of the genes examined, because of the high standard deviation, with the exception of Klf4, whose levels were significantly lower at 5% O_2_ than at 3% O_2_. Nanog levels were significantly higher in iPSC derivation at 5% O_2_ compared with control PBMCs but showed no differences toward 3% O_2_ or normoxia, suggesting that iPSC derivation entails an increase in Nanog levels. Similar results were obtained for OF derivation with respect to Nanog expression, with the tendency, once again, of increased Nanog levels under all the conditions. When comparing iPSC versus OF derivations under the same metabolic conditions, ESRG levels were significantly higher in normoxia-derived OFs. At 3% O_2_, Oct4 and ESRG levels were significantly lower in OFs; at 5% O_2_, lower Oct4, Klf4, and Nanog levels were observed compared with those of iPSCs. Otherwise, Oct4 levels did not differ by condition, because of the high standard deviation, which agrees with previous publications showing that Oct4 levels are critically important for pluripotency and that, in fact, reduced Oct4 expression directs a robust pluripotent state in ESCs [61] and that a higher range of Oct4 levels in ESCs causes heterogeneity in Nanog expression. This concept is also supported by the re-definition of the low Oct4 levels required for pluripotency entry as well as the higher Oct4 levels required for differentiation [19,62].

In vivo studies have shown that lymphoid organs are exposed to an approximately 5% oxygen concentration. Arterial blood contains about 12% oxygen, whereas venous blood varies from 6% to 9% oxygen under normal conditions [63]. At a physiological mixed venous PO_2_ of 40 mm Hg, the O_2_ concentration is 5%. In patients with COPD, 3% oxygen corresponds to in vivo hypoxemia (less than 75% oxygen saturation in venous peripheral blood [64]). These facts could account for the results of our study.

Importantly, we have demonstrated that in vivo physiological hypoxia increases both the reprogramming and oncogenic focus formation efficiencies of at least some peripheral blood cell types. In patients with COPD, the narrowing and destruction of the airways promote generalized hypoxia (hypoxemia) [65] and an increased predisposition to various types of cancer of unknown cause [4]. Our data suggest the direct involvement of physiological hypoxemia in this process. Several findings support our hypothesis that physiological hypoxia could be involved in this oncogenic transformation in vivo: seminal studies have demonstrated that cell metabolism deregulation contributes to oncogenesis [66] and to the CSC phenotype [67]. It is also widely accepted that the development of solid tumors is accompanied by the onset of hypoxia, and hypoxic stress instigates the tumor cell evasion of apoptosis, a central step in tumorigenesis [68]. Although hypoxia-inducible factor manipulation has been extensively studied, but has been only modestly successful in tumor regression [69], several recent findings have suggested that manipulating downstream genes, such as GPRC5A [70], might induce apoptosis in cancer cells by inactivating the GPRC5A-YAP-Bcl2L1 pathway. A major challenge remains in identifying additional molecular targets that hypoxic cancer cells depend on for survival [70,71]. Further investigation is needed into the metabolic programs that support proliferation and the processes by which metabolic states are intimately entwined with the cell-fate decisions that characterize stem cells and cancer cells. The conversion from glucose to lactate, despite the presence of sufficient oxygen to sustain complete oxidation (the Warburg effect or aerobic glycolysis), is a hallmark of all rapidly proliferating mammalian cells. Indeed, iPSCs engage in aerobic glycolysis, consuming large quantities of glucose, and this glycolytic flux is contingent on cells maintaining their pluripotent identity. Mechanistically, the core pluripotency transcription factor, Oct4, binds loci encoding glycolytic genes and, consequently, promotes glycolysis [72] (For a review, see [31].). In addition, there is a biochemical link between metabolites and chromatin-modifying enzymes, leading to the intriguing hypothesis that metabolites exert effects on cell-fate decisions by regulating gene expression programs involved in self-renewal and lineage differentiation [73,74] (For a review, see [31].).

In vivo supplemental oxygenation decreases intratumoral hypoxia and, in turn, improves lung tumor regression in tumor-bearing mice [75], and mitochondrial inhibitors inhibiting the mitochondrial electron transport chain at the bc_1_ complex promote lung tumor regression in humans [76]. The application of hyperoxia is a mainstay treatment for respiratory distress [77]. Our results support the hypothesis that supplemental oxygenation could be used as a cancer-preventive treatment for patients with COPD.

It seems clear, then, that PBMCs are not completely reprogrammable by the methods and factors employed in this study and that certain extra factors are necessary. However, the change in methods/factors employed could mask hypoxia’s role in reprogramming/oncogenic transformation. One possible explanation could be the controversial role of Klf4, an important factor in reprogramming for the maintenance of pluripotency and the immortalization of several cell types [19,43]. However, Klf4 has been shown to be constitutively expressed in monocytes (but not in lymphocytes) and is involved in the differentiation of monocytes from hematopoietic stem cells and myeloid progenitor cells [78]. It is, therefore, possible that Klf4 overexpression in monocytes is interfering with monocyte reprogramming.

This study showed that PBMCs can be used as a cell source both for iPSC formation and OF formation. The former can be used to study various diseases in specific cell types derived from iPSCs from a particular donor and to erase mutations by gene therapy, thereby identifying disease-causing genes. The latter could be used as a tool in the research of oncogenic transformation pathways in diseases associated with a higher incidence of cancer. Both processes could help to investigate how physiological symptoms are affected by cells’ metabolic or epigenetic memory, thereby revealing new drug targets. Given that it has been possible to demonstrate that physiological hypoxia affects oncogenic transformation by increasing its efficiency, another method for deepening our understanding of these processes in patients with hypoxemia could be to establish these lines from other cell types that are easier to reprogram, such as ADSCs.

## Data Availability

No new data were created.

## Supplementary Materials

The following supporting information can be downloaded at https://www.mdpi.com/article/10.3390/cells13110971/s1: Figure S1: Transfection efficiency with respect to cell type and oxygen concentration; Figure S2: Isolation of lymphocytes and monocytes; Figure S3: Colonies of iPSCs and OFs reprogrammed from the peripheral blood monocytes of COPD patients by retroviral or Sendai vectors.
