# Supplementary material for: Hypoxia Increases the Efficiencies of Cellular Reprogramming and Oncogenic Transformation in Human Blood Cell Subpopulations In Vitro and In Vivo
Source: Cells. 2024 Jun 4;13(11):971. doi: 10.3390/cells13110971 (PMC11172288; doi:10.3390/cells13110971)
Supplement: Supplementary file 1 [file cells-13-00971-s001.zip › cells-3023707-supplementary.pdf]

A

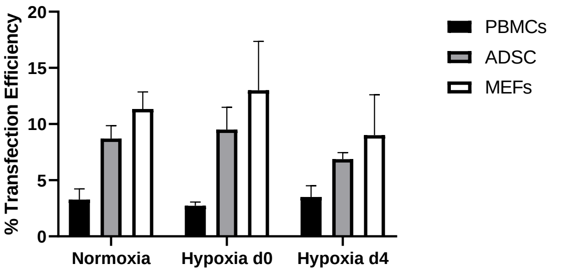


B

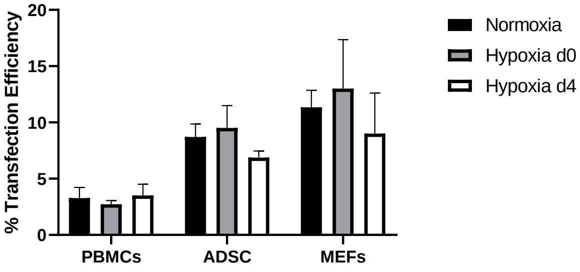


**Supplemental Figure 1. A: Transfection efficiency respect to cell type.** The highest efficiency is achieved for MEFs (white bars) and the lowest for PBMCs (black bars), with intermediate efficiency for ADSC cells (grey bars). **B. Transfection efficiency respect to oxygen concentration.** There are no statistically significant differences within each cell type between the conditions of normoxia (black bars), hypoxia from the day of transfection (grey bars) and hypoxia after 4 days of transfection (white bars).

**MONOCYTE ISOLATION FROM PATIENT 15 PBMCs (male 52 y.o.)**

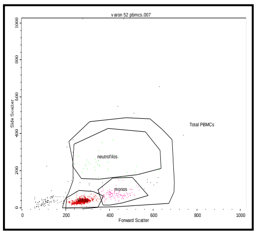


File: varon 52 pbmcs.00
Total Events: 7905

| Region | Events | % Gated | % Tota |
|---|---|---|---|
| linfos | 6474 | 81.90 | 81.90 |
| neutrofilos | 147 | 1.86 | 1.86 |
| monos | 594 | 7.51 | 7.51 |
| Total PBMCs | 7339 | 92.84 | 92.84 |

**LYMPHOCYTES 88%**

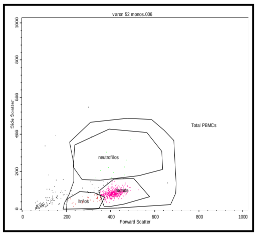


File: varon 52 monos.00
Total Events: 4455

| Region | Events | % Gated | % Tota |
|---|---|---|---|
| linfos | 199 | 4.47 | 4.47 |
| neutrofilos | 64 | 1.44 | 1.44 |
| monos | 3320 | 74.52 | 74.52 |
| Total PBMCs | 3698 | 83.01 | 83.01 |

**MONOCYTES 90%**

**Suppl Figure 2.** Isolation of lymphocytes and monocytes from the blood of a healthy donor. A 90% enrichment in the monocyte population is observed.

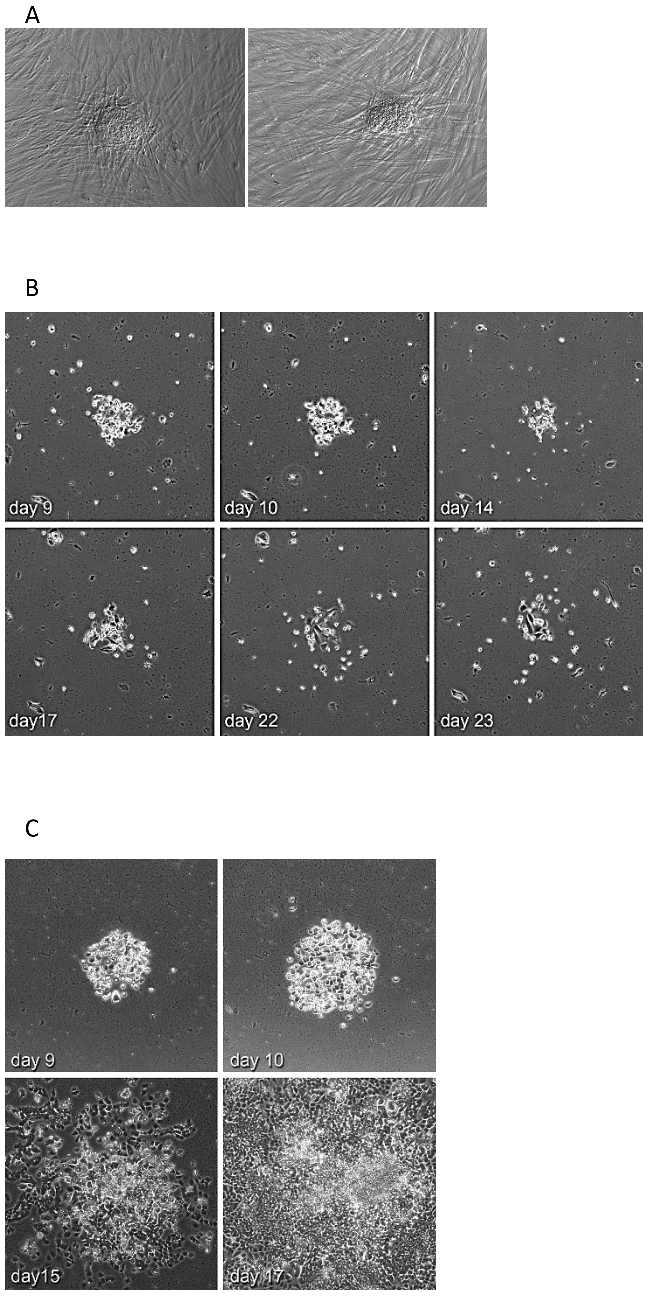


**Supplemental Figure 3. A:** Colonies of iPSC (left) and OF (right) reprogrammed from COPD patient peripheral blood monocytes by retroviral vectors. **B:** CD14 positive cells infected with Sendai Cytotune 2.0. Pictures showing the same colony growing after 9, 10, 14, 17, 22 and 23 days of infection. **C:** CD14negative cells infected with Sendai Cytotune 2.0. Pictures showing the same colony growing after 9, 10, 15 and 17 days of infection.
